# Parental divorce and smoking dependence in Lebanese adolescents: the mediating effect of mental health problems

**DOI:** 10.1186/s12887-022-03523-8

**Published:** 2022-08-04

**Authors:** Vanessa Azzi, Katia Iskandar, Sahar Obeid, Souheil Hallit

**Affiliations:** 1 School of Medicine and Medical Sciences, Holy Spirit University of Kaslik, P.O. Box 446, Jounieh, Lebanon; 2 School of Pharmacy, Lebanese International University, Beirut, 1105 Lebanon; 3 Lebanon Faculty of Pharmacy, Lebanese University, Hadath, 1103 Lebanon; 4 Social and Education Sciences Department, School of Arts and Sciences, Lebanese American University, Jbeil, Lebanon; 5 Psychology Department, College of Humanities, Effat University, Jeddah, 21478 Saudi Arabia; 6 Research Department, Psychiatric Hospital of the Cross, Jal Eddib, Lebanon

**Keywords:** Adolescents, Cigarette Dependence, Lebanon, Parental Divorce, Smoking, Waterpipe Dependence, Depression, Anxiety, Stress, Mental health

## Abstract

**Background:**

Lebanon has the highest prevalence estimates among Middle Eastern countries and Arab women regarding cigarette smoking, with 43% of men and 28% of women involved in such trends. Marital disruption is a tremendous source of irritability and discomfort that may hinder a child's healthy development, creating perturbing distress and increasing disobedience that may exacerbate smoking addiction. Additionally, Lebanese adolescents are inflicted by high emotional and economic instability levels, rendering increased susceptibility to distress and propensity to engage in addictive behavior. This study aims to investigate the association between parental divorce and smoking dependence among Lebanese adolescents, along with exploring the potential mediating effect of mental health disorders of such correlation.

**Methods:**

A total of 1810 adolescents (14 and 17 years) enrolled in this cross-sectional survey-based study (January-May 2019). Linear regressions were conducted to check for variables associated with cigarette and waterpipe dependence. PROCESS v3.4 model 4 was used to check for the mediating effect of mental health disorders between parental divorce and smoking dependence.

**Results:**

Higher suicidal ideation and having divorced parents vs living together were significantly associated with more cigarette and waterpipe dependence. Higher anxiety was significantly associated with more waterpipe dependence. Insomnia and suicidal ideation played a mediating role between parental divorce and cigarette/waterpipe dependence.

**Conclusion:**

Our results consolidate the results found in the literature about the association between parental divorce and smoking addiction and the mediating effect of mental health issues. We do not know still in the divorce itself or factors related to it are incriminated in the higher amount of smoking in those adolescents. Those results should be used to inspire parents about the deleterious effect of divorce on their children to lower their risk of smoking addiction. Further longitudinal studies are needed to better understand the complexity of such associations and to see whether the divorce experience by itself or the factors that accompany it are involved in the increased smoking addiction among adolescents.

## Background

In recent years, health care practitioners and researchers have been interested in the upsurge in psychoactive substance consumption, particularly among young adults. Remarkably, licit psychoactive substances consumption, such as cigarettes and waterpipes, is particularly widespread in adolescence and early adulthood, considered as periods of peak risk for onset and intensification of substance use behaviors that pose risks for short- and long-term health harm [1]. Undoubtedly, tobacco use is a significant public health threat, affecting more than 8 million people worldwide yearly [2], with a rapid increase in developing countries [3], mainly the Middle East region [4]. Accordingly, in 2005, the Global Youth Tobacco Survey, depicted as an all-inclusive, worldwide surveillance of smoking practice among young adults, reported that 8.6% of students smoked cigarettes while waterpipe smoking estimates vastly surpassed those of cigarettes with a 33.9% score [5]. Likewise, previous findings revealed that Lebanon had the highest prevalence estimates among Middle Eastern countries and Arab women regarding cigarette smoking [3], with 43% of men and 28% of women involved in such trends.

Moreover, Lebanon is also a significant region threatened by an escalating waterpipe smoking epidemic, with up to 35% of adolescents aged from 13 to 15 years old extensively using it [6]. Adolescence refers to a tremendously stressful transitional period [7], during which adolescents experience self-discovery to form their identity and interpersonal purposes, biological maturation, mental development processes, and determination for independence while establishing prominent social interplay with one’s environment [8–10]. Consequently, this array of adaptational patterns may trigger adverse emotional conditions [11], such as anxiety [12], depressive symptoms [13], and low self-esteem [14], interfering with their everyday functioning.

Previous studies revealed that adolescents who engage in the smoking practice, are highly likely to be inflicted by significant familial discordance; hence, they tend to be intensively affected by compensatory behaviors such as smoking habits to cope with their mental deficiencies and emotional needs [15–17]. For instance, according to Jessor et al., marital disruption and familial communication are major predictors of problematic behaviors, including alcohol consumption and smoking [18]. As such, several papers revealed that parental divorce impedes mental processes and social interconnections, rendering subsequent engagement in delinquency and deleterious patterns [19–21].

Moreover, previous literature highlighted increased prevalence estimates of adolescent smokers among dissolute families compared to those living with both of their parents [22, 23]. Furthermore, Wolfinger demonstrated that enduring marital disruption as a child dramatically increases the propensity to smoke in adulthood [24]. This might be due to depressive symptoms arising from familial dissolution [25] accompanying familial discordance [26]. However, several findings have established more significant irritability, depression, and emotional stressors stemming from familial discordance, which may trigger smoking and alcohol consumption [27, 28]. Thus, it has been deemed compelling to examine other speculated factors correlated to smoking practice thoroughly.

Several researchers speculated the correlation of substance dependence with mental health disorders such as anxiety, suicidal ideation, and insomnia [29–31]. For instance, it has been shown that smoking prevalence estimates increase with anxiety [30] as an adaptive strategy to attenuate such disturbing feelings [31]. Likewise, other findings revealed that primary smoking subsequently predicted suicidal ideation [32]. Further, Groenman et al. highlighted the fundamental role of implementing diagnostic and interventional strategies to prevent substance dependence in adulthood [33], while other findings demonstrated the efficacy of treating dependence in drastically reducing depression and anxiety symptoms [34].

Since divorce rates are steadily growing in Lebanon (an increase of 101% between 2006 and 2017) [35, 36], and since previous international studies [37, 38] have shown a relationship between divorced parents and adolescents’ addiction to smoking, assessing the background of the Lebanese situation was deemed necessary. Additionally, Lebanese adolescents are inflicted by high mental health [36, 39, 40], and emotional and economic instability levels, rendering increased susceptibility to distress and propensity to engage in addictive behavior [41]. Therefore, it is essential to identify factors incriminated in smoking dependence thoroughly, as such patterns do not hazardously occur among the population. Moreover, to our knowledge, no other study has investigated the potential mediating effect of mental health disorders in the association of parental divorce with smoking among adolescents yet. Hence, it is conceivable that the role of mental health disturbances may have been underestimated and requires further examination. Hence, this study aims to investigate the association between parental divorce and smoking dependence among Lebanese adolescents, along with exploring the potential mediating effect of mental health disorders of such correlation.

## Methods

### Participants

A total of 1810 adolescents out of 2000 approached (90.5%), aged between 14 and 17 years, accepted to participate in this cross-sectional study (January and May 2019). It used a proportionate random sampling of schools from all five Lebanese governorates, according to the list of the Ministry of Education and Higher Education. Out of 18 private schools approached, two declined, and 16 accepted to participate (4 in Beirut, 2 in South Lebanon, 6 in Mount Lebanon, 2 in North Lebanon, and 2 in Beqaa). All students from each school were eligible for participation. They had the right to accept or refuse to enroll in the study; with no financial rewards given in return. The methodology used in this study is similar to that in previous papers [36, 42–47].

### Minimum sample size calculation

Based on the G-power software, a minimum sample size of 395 students was needed to have adequate statistical power for the multivariable analysis, according to 9 factors that should be entered in the final model.

### Questionnaire

The Arabic questionnaire used was anonymous and required approximately 60 min to complete. Students filled it at school to eliminate parents influencing interventions. It consisted of two parts. The first one assessed the sociodemographic characteristics of the participants, their self-reported height and weight to calculate the Body Mass Index (BMI), the number of persons in the household, and the number of rooms in the house, excluding the bathroom and the kitchen, to calculate the household crowding index (the number of persons divided by the number of rooms) [48]. It also collected the self-reported intensity, duration, and frequency of daily activity to calculate the Total Physical Activity Index by multiplying the three factors [49].

The second part included the following scales:

#### Lebanon Waterpipe Dependence Scale-11 (LWDS-11)

The test includes 11 items that are measured on a 4-point Likert scale to evaluate waterpipe dependence [50, 51]. Higher scores reflect higher waterpipe dependence, which was clarified as having a score of ≥ 10.

#### Fagerstrom Test for Nicotine Dependence (FTND)

The test is based on six elements that assess cigarette dependence [52]. The scoring depends on the type of questions; yes/no questions have a score from 0 to 1, whereas multiple-choice questions are scored from 0 to 3. The higher the FTND score, the more intense the physical nicotine dependence. The scale has been previously validated in Lebanon [53].

#### Lebanese Anxiety Scale (LAS)

It is a 10-item tool, created for anxiety assessment among adults [54] and adolescents [55]. Items are scored based on a three- or four-point Likert scale. Higher scores reflect more anxiety.

#### The Adolescent Depression Rating Scale (ADRS)

This 10-item scale was developed to screen for depression among adolescents, with questions rated as yes/no. Higher scores indicate higher levels of depression [56]. Two translators performed a forward and backward translation (English-Arabic-English) for the ARDS scale. Discrepancies between the two English versions were solved by consensus.

#### Columbia–Suicide Severity Rating Scale (C-SSRS)

This 6-item tool, validated in Lebanon [39, 57], is used to assess suicidal ideation and behavior. A score of 0 indicates the absence of suicidal ideation, while a score of 1 or more confirms the opposite [58].

#### Lebanese insomnia scale (LIS-18)

It is made from 18 items that cover all aspects of insomnia and its symptoms, scored on a five-point Likert scale [59]. Higher scores indicate higher insomnia levels.

### Statistical analysis

Data analysis was performed on SPSS software version 25. Cronbach’s alpha values were recorded for all the scales to assess internal consistency. Missing values constituted less than 5% of the total data and thus were not replaced. The FTND and LWDS scores were normally distributed, as verified by skewness and kurtosis values between -2 and + 2 [60]. These assumptions were consolidated by the sample size exceeding 300 [61]. Accordingly, the Student t-test was used to compare two means, whereas the Pearson test was used to correlate two continuous variables. Two linear regressions were conducted, taking the cigarette and waterpipe dependence scores as dependent variables respectively.

The mediation analysis was done using PROCESS v3.4 model 4 [62]. Pathway A determined the regression coefficient for the effect of parental divorce on the mediators (depression, anxiety, insomnia, and suicidal ideation), Pathway B examined the association between the mediators on cigarette/waterpipe dependence, independent of the psychological distress, and Pathway C’ estimated the total and direct effect of parental divorce on cigarette/waterpipe dependence. Pathway AB calculated the indirect intervention effects. Significance was assumed when the confidence interval did not include zero [62]. Independent variables entered in the linear regressions and the mediation analysis models were those that showed an effect size or a correlation coefficient ≥ │0.24│to achieve more parsimonious models [62]. Significance was set at a *p* < 0.05.

## Results

The Cronbach’s alpha values in this study were as follows: LWDS (0.888), FTND (0.825), LAS (0.927), ARDS (0.940), C-SSRS (0.966) and LIS (0.742).

Table 1 summarizes the sociodemographic characteristics of the participants. The mean age was 15.42 ± 1.14 years, with 53.3% of females. Additionally, 11.9% of adolescents had separated/divorced parents. Finally, 408 (22.5%) had waterpipe dependence (scores ≥ 10), whereas 459 (25.4%) had cigarette dependence (scores ≥ 0).

**Table 1:** Sociodemographic characteristics of the study sample

	**Mean ± SD**
Age (in years)	15.41 ± 1.14
Household crowding index	1.00 ± 0.64
Waterpipe dependence score	4.73 ± 8.68
Cigarette dependence score	1.53 ± 2.83
Depression	4.65 ± 2.10
Anxiety	17.88 ± 8.66
Suicidal ideation	1.02 ± 1.84
Insomnia	50.85 ± 12.42

### Bivariate analysis

Higher cigarette and waterpipe dependence was found in adolescents whose parents are divorced compared to living together and was significantly and positively associated with more insomnia, anxiety, depression, and suicidal ideation. Furthermore, it was negatively but weakly associated with older age and a higher household crowding index (Table 2).

**Table 2:** Bivariate analysis taking the FTND and LWDS-11 as the dependent variables

	**FTND**	***p*-value**	**LWDS**	***p*-value**
	**Mean ± SD**		**Mean ± SD**	
**Gender**				
Male	1.50 ± 2.74	0.648	5.00 ± 8.87	0.225
Female	1.56 ± 2.91		4.50 ± 8.51	
Effect size	0.021		0.057	
**Parental status**				
Living together	1.22 ± 2.52	** < 0.001**	4.09 ± 8.37	** < 0.001**
Divorced	3.90 ± 3.77		9.65 ± 9.47	
Effect size	0.835		0.622	
	**Correlation coefficient**	***p*-value**	**Correlation coefficient**	***p*-value**
Insomnia	0.098	** < 0.001**	0.148	** < 0.001**
Anxiety	0.166	** < 0.001**	0.353	** < 0.001**
Depression	0.109	** < 0.001**	0.220	** < 0.001**
Suicidal ideation	0.513	** < 0.001**	0.391	** < 0.001**
Age	-0.147	** < 0.001**	-0.152	** < 0.001**
Household crowding index	-0.089	** < 0.001**	-0.081	**0.001**
Physical activity score	-0.042	0.097	-0.021	0.411

*FTND* Fagerstrom Test for Nicotine Dependence, *LWDS-11* Lebanon Waterpipe dependence scale – 11

### Multivariable analysis

The results of a first linear regression, taking the cigarette dependence scale (FTND scale) as the dependent variable, showed that higher suicidal ideation (B = 0.72) and having divorced parents vs living together (B = 1.21) were significantly associated with higher cigarette dependence (higher FTND scores) (Table 3, Model 1).

**Table 3:** Multivariable analysis

**Model 1: Linear regression taking the FTND scale as the dependent variable**					
**Variable**	**Unstandardized Beta**	**Standardized Beta**	***p***	**95% Confidence Interval**	
Suicidal ideation	0.72	0.46	** < 0.001**	0.65	0.80
Parental status (divorced vs living together*)	1.21	0.14	** < 0.001**	0.82	1.60
**Variables entered in the models**: parental status and suicidal ideation. Adjusted R^2^ = 27.9%, *p* < 0.001					
**Model 2: Linear regression taking the LWDS-11 as the dependent variable**					
Suicidal ideation	1.63	0.33	** < 0.001**	1.40	1.86
Anxiety	0.34	0.32	** < 0.001**	0.29	0.38
Parental status (divorced vs living together*)	2.18	0.08	**0.001**	0.95	3.42

Numbers in bold indicate significant *p*-values; **Variables entered in the model:** parental status, suicidal ideation, and anxiety. Adjusted R^2^ = 26.3%; *p* < 0.001

* indicates significant *p*-values

The results of a second linear regression, taking the waterpipe dependence scale (LWDS-11 scale) as the dependent variable, showed that higher suicidal ideation (B = 1.63), higher anxiety (B = 0.34) and having divorced parents vs living together (B = 2.18) were significantly associated with more waterpipe dependence (higher LWDS-11 scores) (Table 3, Model 2).

### Mediation analysis

Insomnia and suicidal ideation mediated the association between parental divorce and cigarette dependence (Table 4, Model 1) and between parental divorce and waterpipe dependence (Table 4, Model 2).

**Table 4:** Mediation analysis

**Model 1: Cigarette dependence score as the dependent variable**																
	Effect of parental divorce on the mediating variable					Effect of parental divorce and the mediating variable on cigarette dependence					The direct effect of parental divorce on cigarette dependence					Mediating effect of the mediator
	Beta	t	p	95% BCa CI		Beta	t	p	95% BCa CI		Beta	t	p	95% BCa CI		
Parental divorce	-0.43	-0.67	0.502	-1.67	0.82	2.68	13.25	** < 0.001**	2.28	3.07	2.65	12.93	** < 0.001**	2.25	3.05	-
Anxiety						0.06	7.24	** < 0.001**	0.04	0.07						
Parental divorce	-4.71	-5.10	** < 0.001**	-6.53	-2.90	2.85	13.84	** < 0.001**	2.44	3.25	2.70	13.12	** < 0.001**	2.30	3.11	5.06%
Insomnia						0.03	5.63	** < 0.001**	0.02	0.04						
Parental divorce	2.05	15.92	** < 0.001**	1.79	2.30	1.27	6.40	** < 0.001**	0.88	1.66	2.74	13.27	** < 0.001**	2.34	3.15	115.08%
Suicidal ideation						0.72	20.00	** < 0.001**	0.65	0.79						
Parental divorce	-0.29	-1.86	0.063	-0.59	0.02	2.72	13.40	** < 0.001**	2.32	3.12	2.68	13.12	** < 0.001**	2.28	3.08	-
Depression						0.15	4.59	** < 0.001**	0.08	0.21						
**Model 2: Waterpipe dependence score as the dependent variable**																
	Effect of parental divorce on the mediating variable					Effect of parental divorce and the mediating variable on waterpipe dependence					The direct effect of parental divorce on waterpipe dependence					Mediating effect of the mediator
Parental divorce	-0.43	-0.67	0.502	-1.68	0.82	5.65	9.37	** < 0.001**	4.47	6.83	5.50	8.51	** < 0.001**	4.23	6.76	-
Anxiety						0.36	15.55	** < 0.001**	0.32	0.41						
Parental divorce	-4.72	-5.11	** < 0.001**	-6.53	-2.90	6.16	9.53	** < 0.001**	4.89	7.42	5.59	8.60	** < 0.001**	4.32	6.87	9.19%
Insomnia						0.12	7.04	** < 0.001**	0.09	0.15						
Parental divorce	2.05	15.92	** < 0.001**	1.79	2.30	1.91	2.88	**0.004**	0.61	3.21	5.53	8.43	< 0.001	4.24	6.81	189.74%
Suicidal ideation						1.77	14.83	** < 0.001**	1.54	2.00						
Parental divorce	-0.29	-1.86	0.064	-0.59	0.02	5.86	9.32	** < 0.001**	4.62	7.09	5.60	8.72	** < 0.001**	4.34	6.86	-
Depression						0.89	8.95	** < 0.001**	0.69	1.08						

## Discussion

Our results showed a percentage of 22.5% for waterpipe dependence and 25.4% for cigarette dependence among enrolled students. Those numbers are higher than the ones reported by Bejjani et al. in 2012 (3.9% frequent cigarette smokers and 19% frequent waterpipe smokers [63]). Adolescents are at higher risk of substance experimentation, particularly if that substance is obtained easily and if their surroundings approve of such behavior [10]. They highly tend to search for novelty and reward dependence, while less avoiding harm [64]. Neuromaturational changes may explain the reasons for negative health behaviors seen among adolescents (substance use/abuse, mental health issues, and violence) [65]. During this complex phase, adolescents face biological and experiential changes and chart their lifestyles, which will have long-term impacts on all facets of their growth, including health [66]. These substances are often associated with partying, socializing, sharing, new experiences, and breaking the prohibitions characteristic of the adolescent period [67]. Young people do not seem to be aware of the dangers that these substances represent to their health: at their age, they display a cerebral immaturity which results in the neglect of these risks, reinforced by the fact that the damage is often observed on the means and the long-term [68].

Our findings revealed that parental divorce was significantly correlated with licit psychoactive substance consumption (cigarette and waterpipe), in line with previous literature [22, 23]. Parents neglect their children at the beginning of the divorce (while trying to resolve their issues); thus, children have a higher tendency to search for such substances to fulfill their emotional needs [69]. According to social learning theory, children's social behaviors are highly afflicted by parental warmth and interactions they witness within their families [70]. Adolescents turn to cigarettes and water pipes as a means to get the attention of their parents and as a means of “self-medication” without thinking about the long-term harmful effect of smoking [71].

Our results showed that the association between parental divorce and cigarette/waterpipe dependence is mediated by mental illness (insomnia and suicidal ideation). The stress experienced by the child during the divorce period creates an unhealthy home environment, which might consequently have a negative effect on their health [17]. Psychological distress, anger, and depression in adolescents that accompany the divorce period [22, 27, 28, 72, 73], along with a high sensation of revolution, may trigger the initiation of risky behaviors such as nicotine addiction and alcohol use [74, 75]. Previous results suggest that anxious people tend to smoke more [76]. This specifically applies to adolescents who experience conflicts within their families and turn to smoke to satisfy their psychological needs [15]. Waterpipe is becoming more popular among adolescents [6, 77] since they are more tempted to smoke water pipes because of the different flavors [78], and the lower knowledge and worse attitude about waterpipe and its harmful effects [79–82].

The current political and economic instability in Lebanon, in addition to the displacement of many Syrians following the war in their country [83], are additional causes of mental illness and destabilization among Lebanese adolescents [41, 84] and adults [57, 85–87]. Furthermore, Lebanese adolescents have low civic engagement and belonging to their country and feel desperate from the country’s political system following the low budget allocation, and the absence of implementation of policies and programs for youth [41].

### Clinical implications

Our results have several implications. Divorcing parents need to be aware that their problems might reflect on their children's use of substances [72]. Parents should work together to make this period as easy as possible for the child and try to work with counseling services aiming at reducing parent–child conflict during parental divorce. In fact, adolescents who spent more time with their parents had less smoking [88]. Healthcare professionals should do their best to identify adolescents with mental health issues and who also have a tendency for substance use/abuse, which might prevent and treat consequent problems from the use of addictive substances [89].

### Limitations

The study is cross-sectional, and thus, does not permit the inference of causality between variables. A selection bias is present since students from public schools and adolescents who do not attend schools are not represented in our sample. The symptoms were self-reported by students and were not evaluated by a healthcare professional, predisposing us to an information bias. A residual confounding bias is present as well since not all factors associated with addictions were taken into consideration in this study (parental psychiatric diseases, alcohol, smoking, and drug abuse). Adolescents were not asked when they started smoking and when their symptoms (for example insomnia) were observed (before or after the divorce).

## Conclusion

Our results consolidate the results found in the literature about the association between parental divorce and smoking addiction and the mediating effect of mental health issues. We do not know still in the divorce itself or factors related to it are incriminated in the higher amount of smoking in those adolescents [88]. Those results should be used to inspire parents about the deleterious effect of divorce on their children to lower their risk of smoking addiction. Further longitudinal studies are needed to better understand the complexity of such associations and to see whether the divorce experience by itself or the factors that accompany it are involved in the increased smoking addiction among adolescents.

## Data Availability

The datasets generated and/or analyzed during the current study are not publicly available due to restrictions from the ethics committee but are available from the corresponding author (SH) upon reasonable request.

## Abbreviations

WHO: World Health Organization
LWDS: Lebanon Waterpipe Dependence Scale
FTND: Fagerstrom Test for Nicotine Dependence
LAS: Lebanese Anxiety Scale
ADRS: Adolescent Depression Rating Scale
C-SSRS: Columbia–Suicide Severity Rating Scale
LIS: Lebanese Insomnia Scale
